# An Activity Tracker and Its Accompanying App as a Motivator for Increased Exercise and Better Sleeping Habits for Youths in Need of Social Care: Field Study

**DOI:** 10.2196/mhealth.9286

**Published:** 2018-12-21

**Authors:** Kari Rönkkö

**Affiliations:** 1 Department of Design Faculty of Business Kristianstad University Kristianstad Sweden

**Keywords:** mHealth, social work, youths, activity trackers, mobile applications, motivation, self-care, sleep hygiene, goals

## Abstract

**Background:**

The number of mobile self-tracking devices connected to the Web has exploded in today’s society. With these wearable activity trackers related to Web 2.0 apps and social media have come new ways of monitoring, measuring, representing, and sharing experiences of the human body. New opportunities related to health and new areas of implementation for professionals have appeared, and one identified area that can benefit from mobile health technologies is social work.

**Objective:**

There are still only a small number of papers reporting the results from studying wearable activity trackers and accompanying apps in the context of agency-based social work. This study aimed to contribute to the identified shortage by presenting results from a research project framed by the following overarching question: *What effects will the studied youths in need of social care experience in relation to exercise and sleep as the result of using a wearable activity tracker and its accompanying app?*

**Methods:**

A field study framed by action research was performed. The study concerned vulnerable youths living in a Swedish municipality’s care and accommodation home that tried out an activity tracker and its accompanying app.

**Results:**

The results from the study confirm previously published research results reporting that instant graphical feedback, sharing information, and being part of a social community can have a positive impact on lifestyle changes. In addition, this study’s main results are that (1) the most important factor for positive health-related lifestyle changes was the establishment of personal long-term goals and (2) professional social workers found the studied technology to function as a valuable counseling tool, opening up avenues for lifestyle talks that otherwise were hard to undertake.

**Conclusions:**

This study demonstrates how an activity tracker and its accompanying app can open up a topic for discussion regarding how vulnerable youths can achieve digital support for changing unhealthy lifestyle patterns, and it shows that the technology might be a valuable counseling tool for professionals in social work.

## Introduction

Over the last 10 years, mobile devices have become widely adopted in society. Today, small, portable digital devices are widely spread and able to connect remotely to the internet from most locations. Within the practice of medicine and public health supported by mobile devices, so-called mobile health (mHealth), there follow many promising developments. The option to use mobile technology to collect data on one’s bodily functions and everyday activities has received much attention [1-4]. Frequent statements have also been made in the popular media and in the medical and public health literature about a revolution in health care, preventive medicine, and public health driven by the use of mobile technologies [5]. Mobile technologies have the capacity to extend the body by supplying data that can be used to display both limits and capabilities, and they allow users to employ the data to work upon and improve themselves. Accounts of self-tracking technologies for health tend to place emphasis on the potential for the empowerment of laypeople and on the importance of taking responsibility for one’s own health [5].

Previous studies have demonstrated that various technologies for health-promoting behavior allowing the user to measure and monitor their own behavior are promising [6-8]. It is also claimed that the concepts of health and health care are moving toward the notion of personalized preventive health and that personalized solutions could be the answer to solving public health challenges at their causal root [1]. It is clear that regular doses of physical movement have a positive influence on people’s health and also that mHealth technology can help motivate people to move more. In relation to exercise by prescription, researchers have asked for more empirical studies on how apps can provide motivation [9]:

I think we have reached the next generation of studies now. They should be about how we get this to work for prescribers and patients. Perhaps patients can get the motivation, support, and monitoring via apps or other technical systems, which gradually give them the results showing that the training is paying off because this is strongly motivating. This is what we should study now when there exists evidence that supports exercise by prescription.

Obviously, it has been believed for some years now that mHealth technologies have the potential to contribute to improved health through increased motivation and changes in bad habits.

Another interesting area besides exercise for mHealth technologies within social work is to provide feedback on bad sleeping patterns. Good sleeping habits can influence the ability to both learn and perform in society. Insufficient sleep is associated with a large number of problems and adverse health behaviors, that is, physical distress, frequent mental distress, activity limitations, depressive symptoms, anxiety, and pain [10]. Having adequate tools, methods, and knowledge to provide increased awareness of sleeping patterns [11] would benefit both people in need of care and professionals within health care and social work. In a systematic literature review published in 2017, evidence was presented that mobile phone interventions have the ability to attenuate sleep disorders and to enhance sleep quality. The same study also claimed that mobile phone intervention methods could provide better sleep solutions in comparison with other recognized treatments such as cognitive-behavioral therapy for insomnia [12].

The present author published results from a literature study in 2017 covering the state-of-the-art research surrounding motivation, wearable activity trackers, and accompanying apps in social work [13]. The main results are summarized here. On the basis of a bibliometric and literature study, it was demonstrated that there are relatively few research connections between concepts that are related to health apps and self-care versus motivation and social work. It seems that different journals have different interests in activity trackers and accompanying apps, for example, they are interested in the clinical, medical, user-oriented, interaction-oriented, management, or technical aspects of the technology. The strongest identified link-based relations between research areas were found in the borderland of medicine and computer science, which largely represented technical discussions of digital solutions in the health area. Connections to social work appeared to be isolated between psychology, psychiatry, and social psychology and also between systems-oriented computer science and user-oriented informatics. In relation to shared concepts between the research areas, it was found that the main focuses were on technology and technical systems; communication technologies and distance treatment; technical design; technology and medical treatment; data security; and privacy issues from technical, political, and legal points of view. In summary, the bibliometric study demonstrated that there are relatively few research connections between concepts that are related to health apps and self-care, motivation, and social work.

The literature review presented in the same publication [13] showed that health apps as a phenomenon had attracted a wide range of researchers from several disciplines. In general, high hopes were expressed for mHealth technologies, and self-care seems to be a growing area and a target for both health and industrial actors. It was believed that eHealth services could empower users to better manage their own health, and the main identified themes were related to digital technology and weight loss, psychological treatment, rapid technical developments, missing professional health competencies during the development of health technologies, and how to handle user data and integrity. Many of the papers focused on the technology itself rather than motivational work, health aspects, and social work. A systematic literature review by Ridgers et al [14] found that there is a paucity of research concerning the effectiveness and feasibility of activity trackers and accompanying apps as tools for increasing children’s and adolescents’ physical activity levels. There is a lack of contributions regarding the combination of sleeping habits, motivation, increased exercise, and social work [13]. There is also a limited amount of information about how mHealth technologies can sustain changes in health behavior and how they can be integrated into health care [15]. There seems to be room for more research regarding how to increase nonactive people’s physical movement and how to improve their sleeping patterns with the help of activity trackers and accompanying apps in the field of social work.

Social work is lagging behind when it comes to adopting these technologies, and researchers have requested more empirical studies of these technologies in the context of social work [16-18]. Identified reasons for the lagging situation are a lack of technological training in the majority of social work degree programs, a lack of professional standards that define technological competency, and concerns that technology might interfere with the relationships on which social work is based [16-18.]. It has also been shown that the vulnerability of the clients in social work requires special ethical consideration when new digital technology is introduced [19]. The lagging also includes the quantified self, for example, with the help of wearable activity trackers and accompanying apps [16]. The identified gap in knowledge is claimed to slow down the adoption of professional knowledge related to information technology [18,16,20]. Furthermore, it is suggested that harnessing technology in the context of social work will allow for more effective service development, planning, and delivery [21]. This paper contributes to the above by presenting results from a 4-month field study of how a wearable activity tracker and its accompanying app were perceived by 8 young people living in care and accommodation homes.

Despite the above-identified lack of rigorous research and conclusive results in this study’s subject of interest, it was still possible to identify useful lessons that were applicable to this study’s area of focus. These included findings that iterative feedback cycles between users (clients), health professionals, and researchers (as inventors) are necessary to successfully address real-world needs [22,23]; that privacy and users’ attitudes are challenges that need to be considered early in the development of the technology [24-27]; that setting goals involving wearable activity trackers and their accompanying apps is motivating [6]; that instant gratification and graphical feedback are especially rewarding [7,28]; and that being part of a social community with friendly competition is motivating and might lead to increased physical activity [28].

This paper presents results from a field study framed by action research. The study aimed to better understand how a specific wearable activity tracker and its accompanying app might influence the motivation of vulnerable youths to exercise more and to improve their sleeping habits in the context of agency-based social work. The study’s results suggest that social workers’ counseling of youths can benefit from shared data from a wearable activity tracker and its accompanying app. The principal result of this paper is increased knowledge of how a wearable activity tracker and its accompanying app can open up for discussions of how activity and sleep patterns among vulnerable youths can be addressed in the context of agency-based social work.

## Methods

### Field Study Setting

The empirical investigation took place in cooperation with Helsingborg, the eighth largest city in Sweden with more than 100,000 inhabitants in its urban area. Helsingborg’s interest in participation in the project was anchored in goal 7 of the city’s strategic vision, that is, the city of Helsingborg will be a leader in utilizing digitization opportunities. They expressed 3 main reasons for their involvement in this study: (1) to learn more about new potential digital technology for preventive social work, (2) to identify new tools that could increase motivation for positive lifestyle changes, and (3) to better understand the potential of the *coolness factor* among children and youths. In comparison with previous research projects in the municipality, this project targeted vulnerable groups with a more complex composition of problems because of weakened or obstructed parenting. In relation to the target group, the municipality also expressed fear that the technology might create new types of problems among the youths. One fear was that the youths might feel obligated to participate and thus reveal habits and thoughts about themselves to their peers, which could be used against them. A second fear was that the technology might interfere with the relationships between clients and professionals on which social work is based. Another question they had was regarding how social workers would be able to identify and try out tools for measuring the health of youths with social problems within social care without being health professionals themselves.

In this project, young people living in care and accommodation homes (Home for Care or Living, HVB-home) in Helsingborg municipality were studied. From an organizational viewpoint, the studied HVB homes were under the social administration and targeted young people aged 13 to 20 years with social and psychosocial problems such as relationship problems, school problems, and incipient risk behavior that might lead to crime. Young people with active addiction are not allowed in the HVB homes. The HVB homes are open accommodations where young people are voluntarily placed, and the voluntary nature of the arrangement makes it easier for the staff to work with the youths. In the HVB homes studied for this project, there was room for 8 youths in a shared accommodation, 9 youths in training apartments, and 2 more youths at 2 ordinary homes. The training apartments were spread out in different neighborhoods in Helsingborg city. The joint youth accommodation consisted of a large house where the young people had their own room, including a large common social area and a shared kitchen and dining area.

The wearable activity tracker Jawbone UP24 (Jawbone Limited) was used in the project. The activity tracker synchronized with an app (the accompanying app) that had to be downloaded to the user’s own smartphone or tablet. The app recorded the participant’s daily physical activity and sleep patterns, which also could be followed over time. The physical activity was measured by counting the number of steps taken, and sleep was measured by the number of slept hours, that is, the number of hours of light and deep sleep. Jawbone UP24 allowed the users to set reminders, individual step and sleep goals, wake-up alarms, and other alarms. In the app, users could also form teams and follow each other’s results and communicate in a chat forum. More information about Jawbone UP24 that is beyond the scope of this paper can be found in the study by Swider [29].

### Framework for Cooperation and Study

As an overall framework for cooperation and research, the project applied action research [30,31]. This framework allowed us to not only study the social phenomena of common interest but also to jointly alter the object of study in close cooperation cycles. With this approach, we achieved a deeper joint understanding of those aspects that influence the situation of the observed target group at the same time as we could introduce improvements. We implemented a cyclical process of moving from observation to planning, to implementation, and back to observation (various examples of implementations that are beyond the scope of this paper, including some of the author’s own previous experiences, can be found in the study by Dittrich et al [32]). The entire project, including planning, bibliometrics and literature study, implementation of the field study, and joint presentation of the results to the municipality, took place in the period of September 2014 to June 2015. An important goal for the municipality was to achieve hands-on experiences based on academic knowledge. Thus, Helsingborg searched for approaches that could be spread in the municipality’s organization and owned by the social workers themselves. A management and cross-municipal unit responsible for developing methods to improve the conditions for children and young people at risk continued the implementations based on this project’s results, and when taking a retrospective look at the continued nonacademic implementations by Helsingborg, it was found that the academic study results were confirmed (more information about Helsingborg’s continued implementations can be found in the study by Danielsson et al [33]).

### Field Study

In the field study, we addressed the following research question: *What effects will the studied youths experience in relation to exercise and sleep as a result of using a wearable activity tracker and its accompanying app?* We chose to focus on interviews influenced by an ethnographic standpoint, meaning that we emphasized understanding the studied people’s own point of view [34]. We wanted to know how recent digital technology in the form of an activity tracker and accompanying app could support users’ needs and at the same time satisfy the municipality’s ambitions regarding social work. The staff at the HVB homes did not want to be responsible for running the project because of previous negative experiences of project ownership. All the young people and staff in the HVB homes were offered the chance to participate. Because this was the first related project in Helsingborg, we could also offer the youths the chance to be involved in the choice of what type of wearable activity tracker and accompanying app should be used in the project. The field study for this group was conducted from November 2014 to February 2015. The period was planned deliberately to enable investigation of whether there might be a difference in young people’s physical activity and sleep habits during the Christmas holidays. The youths were offered 2 follow-up meetings that were entirely voluntary, and 2 or 3 youths participated in each meeting. Besides these meetings, there was continuous communication with the youths via individual physical meetings, email, short message service text messages, and phone calls. The staff was followed up through group meetings and individually at the end of the project period.

We are aware that the choice of field methods could lead to an inherent bias in the kind and depth of information that can be obtained from the different communication channels, that is, the participant’s personality and willingness to share input. Therefore, we worked hard to establish an atmosphere of mutual trust guided by ethical advice from ethnography [34]. Another potential bias was that the participants on their own initiative decided to introduce a step competition during the second month. They divided themselves into 2 teams and started to compete for a month’s time. Hence, it was difficult to know what role the competition as such might have had on the youths’ motivation during this month. Was it the competitive game or the technology that provided the motivation? One plausible interpretation is that the technology as such acted as an enabler that inspired them to come up with the idea, allowed them to make their results visible to all the participants in the competition, and provided an option to follow up on each other’s results both continuously and historically. Our interpretation was that the technology became a tool for supporting the youths’ ideas and desires, where the instant gratification and graphical feedback of achieved results played an important role for the level of motivation.

The field study included 8 youths aged 17 to 18 years and 12 staff, all of whom had access to the Jawbone UP24 wearable activity tracker linked to a smartphone or tablet with the accompanying app installed. The wearable activity tracker was synchronized with the accompanying app that was downloaded to the users’ smartphones or tablets. The accompanying app recorded the participants’ daily physical activity and sleep patterns, which could be followed over time. The physical activity was measured by counting movement or number of steps, and sleep was measured by counting the number of slept hours divided into hours of light and deep sleep. In the app, the user could set reminders, individual goals, and wake-up alarms. The users could also form teams, follow each other’s results, and communicate with invited friends in a chat forum. The app also included a cost function, which allowed the users to record their daily food intake. This nutritional function was not included in the project because of a lack of expertise in the field of nutrition and also the risk of triggering eating disorders. The project had digital access to allow the researchers to follow all included participants’ results regarding both daily steps and nightly sleep. This material was regularly compiled into an Excel file that was sent out to all members of the research group and that was taken up and discussed during the regular meetings of the research group.

The professional social caregivers played an important role in the project because they were the ones delivering therapy and advice about life to the youths at the shared accommodation. All caregivers voluntarily decided to use the wearable activity tracker and the accompanying app during the project, but their results from using the technology were not something that we registered. The choice to provide the same technology to the caregivers was important because it increased their commitment and understanding of what was being studied. During the implementation of the project, there were continuous informal meetings and conversation occasions with both the staff and the youths, so-called field observations. Data from many of these occasions confirmed the results from the interviews that we present in the Results section. These conversations and informal meetings were an inevitable and natural part of the project implementation related to the project’s follow up of how the technology worked, and they provided understanding of the technology’s usage as well as occasions for educating about the available features in the technology.

A 1-hour group interview was conducted with the staff at the youth HVB home, and 4 individual interviews were held with young people living at the same accommodation. We conducted open interviews focusing on the youths’ own views and experiences, keeping the purpose of the study in mind during the interviews. Each individual interview lasted around 30 min. One of the youths was interviewed over Skype because of having moved abroad. All interviews were recorded and transcribed. The selection of the interviewed respondents was based on the youths’ availability and their willingness to participate because some of the youths were not comfortable with being formally interviewed. The interviews aimed at gaining a deeper level of knowledge regarding the perceived everyday experiences of using the wearable activity tracker and the accompanying app. A central subject in the interviews was whether and how the young people themselves felt that the activity tracker and accompanying app had influenced their situation regarding physical activity and sleeping patterns. Additional information that is beyond the scope of this paper can be found in the project report by Rönkkö et al [13] (in Swedish).

## Results

### Field Study Results

During the first 2 months, November and December, all 8 participants were physically active. Up to the Christmas holidays in late December, there were only minor breaks (a few days) during which some of the youths had not used the technology. The reasons for not using the device were because of illness or that they forgot to put it on again after taking a shower or after charging the activity tracker. After the Christmas holidays, only 4 of the participants were still using the activity tracker, and at the end of the project, only 3 were using the activity tracker and accompanying app. Although not all the participants were positive initially, all the interviewed participants expressed that they experienced increased motivation to exercise as a result of using the activity tracker and accompanying app. Table 1 shows the number of steps that the participants took on average per day during the entire study. Days when no data were recorded were not included and therefore did not affect the results.

Instant gratification and graphical feedback of specified goals were reported to be important, and the possibility to set up individual targets in the accompanying app together with instant gratification and graphical feedback of results motivated the participants to increase their physical activity. The average number of steps was consistently a bit over 10,000 steps per day, which can be considered good because this corresponds to about 8 km daily walking, a result one would expect from active people. Some comments in this regard were:

I’m proud of myself when I see that I have taken so many steps—it was motivating to see how many steps I had taken the last 7 days.

Yes, that is what kept me going, it sort of motivated me to continue and it definitively also helped me in my physical development.

For me it was motivating that we could see each other’s results, to see each other’s results triggered us all.

We could also identify the importance of setting attainable goals that could be increased as the individual’s physical and mental strength increased. Comments in this regard included:

Yes, it motivates me to do more, to move more each day because I always want to reach my daily goal

...

Sometimes when I see, oh shit, I’ve only walked 8,000 steps...but I can still go an extra round.

All the youths reported trying to increase their physical activity during the project. Three of the youths started training at the gym during the project, 1 of them began taking walks with friends to a greater extent than before, and 1 began going by bicycle to school instead of taking the bus as well as taking walks during breaks:

I do not sit during breaks like before; instead, I choose to walk.

Long-term goals and social attention from peers and the project influenced the youths’ motivation. The study period was placed deliberately with a holiday break in the middle to explore what happens to participants’ activity level after a 2-week break. The activity levels were plummeting for 4 of the participants who did not start using the activity trackers again after the Christmas break. We found that half of the participants stopped using the activity tracker and accompanying app during the Christmas break when the social attention from the project and their co-users was low. Only the most dedicated youths maintained their use of the device and app. An investigation revealed that the 4 participants who continued to use the technology had established their own long-term goals. The participants without long-term goals stopped using the technology during the holiday break. We concluded that social attention and having long-term goals were the 2 most important factors for motivation.

Social attention from their surroundings influenced the youths’ attitudes toward technology and toward their health. We found that 1 participant often wore his activity tracker even when the battery was dead; however, at the same time, we learned that he spoke warmly about the benefits of the bracelet with many different people without really being a dedicated user. After closer investigation, we discovered that he found the social attention that came with the activity tracker stimulating. The remaining youths in the project confirmed the first nondedicated youth’s experience by expressing how the project as such gave rise to positive social attention from their surroundings. They expressed that people were often curious to know more about the wearable activity tracker and the connection to health.

Friendly competition from peers was found to be motivating, although taking the competitive element too seriously could become stressful. The youths expressed how they were motivated by the project because it legitimized a social information-sharing culture among the youths. Four of the youths said that they gained increased strength by being part of a team where they could follow each other’s developments and could encourage each other. Comments in this regard included:

For me, it was motivating that we could see each other’s activities.

Being in a team motivated me.

We encourage each other through the sharing of results.

**Table 1:** Average number of steps per day.

Months	November	December	January	February
Participants	8	8	4	3
Steps/day	12,204	10,799	11,523	12,854

It became clear to us that this type of friendly competition within a team played a key role in keeping up the participants’ motivation and activity level. However, we also found that sharing goals within a team could lead to stress. Overall, 4 of the participants felt that it was stressful to see how physically active the others were in the app. Two of them were not pleased with their own results, although they expressed that they were more physically active than they had been before:

I’m not satisfied with my performance, and I’m also stressed by the others in the app.

The stress became even more apparent when a step competition was held between teams, a proposal that came from the participants themselves. The youths divided themselves into teams and started to compete. Two of the youths said that it was hard to follow how many steps their teammates took and to contribute to the entire team’s performance:

I have been sick, and this gives me anxiety because I’m not able to practice and contribute.

Hence, they expressed an experience that leads to increased stress and diminished motivation.

The awareness of one’s own sleeping patterns as a motivator for change was found to be more challenging than the awareness of one’s own activity level as a motivator for change. It was obvious that the activity tracker and app provided useful feedback by visualizing patterns and habits in relation to sleep. Positive comments from the youths were:

It’s a “coach” that tells me when I should go to bed—I have tried it, and it works.

I try to sleep well and it helps me—I wake up easier and have the enough sleep.

I feel clearer in my head now—I sleep better and feel more alert during the day.

Overall, 5 youths had used the alarm function and were satisfied with it. It woke them up in the light sleep phase, whereby they experienced that it was easier to wake up feeling clearer in the head. Three youths had used the reminder function, notifying when it is time to go to bed, and said that it helped them. In addition, 1 youth used the reminder function to remember to take her vitamins, which also worked well. One negative comment was:

I want to the alarm to wake me up, but I do not notice the alarm.

A more problematic comment was:

Now I know how I’m sleeping, but I cannot do anything about it.

The last problem shows that the technology in itself did not generate solutions or provide useful enough information that could change the poor sleeping situation. It was a too difficult a challenge for the youths themselves to figure out what was needed on a practical level to establish a positive change. Going to bed earlier did not necessarily lead to more sleep if there were more complicated reasons behind the sleeping problems. Changing sleeping habits was not as straightforward as going out and walking the 1000 or 2000 steps that were needed to reach a preset goal of 10,000 steps. Our conclusion was that supplementary practical support by qualified professionals was needed in these cases.

Being aware of one’s own habits had a positive value, but it was not enough to lead to sustainable change for all the youths. All participants expressed increased awareness of their own physical activity levels and sleeping habits. In relation to the 4 participants who took off the activity tracker during the Christmas break, we found that this awareness was still valuable for them. Some of the participants said that they felt comfortable with the awareness that they had achieved and the knowledge that they could form decent habits of physical activity if they wanted to. Two of the participants who had taken off the activity tracker expressed that they were more aware of their habits now, but they could not change either their sleeping habits or their attitude toward long-term exercise:

I’m more aware of my actual habits now, but I’m still not able to change the habits; I know more about myself today, but I still cannot change my situation.

Not being able to change their habits after 2 months influenced their decision to stop using the activity tracker during the Christmas break.

The wearable activity tracker and its accompanying app were identified as a potential tool by the caregivers. We experienced a genuine interest among the staff to constantly improve their work and relationships with their clients. The staff in the HVB home had also previously asked their management for tools that could provide access to and improve their possibilities to influence young people’s exercise and sleeping habits. The reason was that the staff had seen so many negative effects of moving too little and of getting poor sleep. At the end of the project, the staff expressed that the activity tracker and accompanying app could be such a tool. The staff expressed a clear value in being able to use this form of technology in their treatment work regarding the youth’s habits and health. They saw a potential to support the creation of structure in young people’s everyday lives:

We usually give advice to young people about picking up their clothes in the morning, eating before bedtime, not watching television just before bedtime, etc. This is a completely different way, to now and then meet the youths and talk about the results from the app.

It became clear that the graphic results from the accompanying app could be used in conversations with the youths to motivate or to concretize problems as well as to raise a topic of conversation about sleep and exercise more generally:

It is good for getting direct feedback that can be used in counseling.

The staff expressed that the activity tracker and the accompanying app had opened up for a new type of talks about habits and health with the youths. The staff also told us that the technology had opened up for similar discussions between the employees themselves because they did not always touch upon these difficult subjects in their own group. One identified troublesome view here was that not all of the staff could claim to be good role models based on their own physical activity levels or sleeping habits. In any case, the staff perceived the activity tracker and accompanying app to be positive instruments for making visible the youths’ habits, which enabled good discussions. To conclude, the staff experienced that the activity tracker and accompanying app could be used with youths for a certain period to increase their awareness and ultimately also their motivation for improving their physical activity levels and improving their sleeping habits.

## Discussion

This study demonstrates how the activity tracker and its accompanying app can open up a topic for discussion regarding how poor lifestyle patterns among vulnerable youths in the context of agency-based social work can be addressed with the help of digital technology. Previous research on health apps to motivate physical activity have demonstrated that the most central factor for success was to include various forms of specific goals that individuals could work toward [6]. In relation to goals, instant gratification and graphical feedback are identified as key elements for motivation [7,8]. These are success factors that correspond well with our study’s results. The studied youths in this project went from being nonexercising to walking around 8 km a day, a result one would expect from active people. Instant gratification and graphical feedback provide one explanation for the success. Additionally identified explanations in this project relate to social attention, long-term goals, and the possibility to adjust goals over time. Success in this project means that the technology helped to motivate 3 of the youths to exercise more to the end of the project and that all youths became more aware of their own physical activity levels and sleeping habits.

The study period was arranged deliberately with a holiday break in the middle to explore what happens with the activity level after a 2-week break. During the break, the youths visited their families. It was found that half of the participants stopped using the activity trackers when the social attention from the project and their co-users was lost. In this study, the youths experienced increased strength because of being part of a team where they could follow each other’s developments and could encourage each other. In previous research, it has been demonstrated that *friendly* competition can increase the motivation for being physically active [28]. The studied youths talked about the social group and the internal competitive games as triggers for activities. The loss of participation took place when the social situation changed. With the lack of motivating social attention, the activity tracker and accompanying app lost their value for half of the studied group. We thus conclude that the 4 youths who stopped using the device over the Christmas break had been motivated primarily by the positive social attention from using the device. After the break, these 4 youths did not find enough motivation to start the social activity again.

Another interesting finding in relation to social attention was that 1 of the participants often wore his activity tracker in discharged mode. Without being a dedicated user, he spoke warmly about the benefits of the bracelet with many different people. This can be explained theoretically by the phenomenon called *ticket to talk* [35]. Ticket to talk is a phenomenon that opens up and legitimizes conversations between people who are unknown to each other; for example, if 1 person passes by an unknown person with a dog, the first person might start a legitimate conversation by commenting that the dog is cute. The ticket to talk, in this case, the cute dog, thus legitimizes 1 stranger starting a conversation with another stranger. After the opening question via the ticket and a first positive response, it is legitimate to talk about dogs in general and thereafter slowly enter other areas that would not have been legitimate to address directly between 2 strangers (different types of contexts and examples can be found in the study by Svensson and Sokoler [36]). In our case, the activity tracker constituted a ticket to talk regarding both health aspects and cool technology (one of the municipality’s expressed interests). The fact that activity trackers were still sort of unusual when starting the project, and were visible, gave both unknown people and the youths themselves legitimacy to start up conversations about, for example, the activity tracker and its connection to exercise and health. People in the surroundings were curious and keen on knowing what the participant had on their wrist and how it worked. Interestingly, the youth in question was not a serious practitioner of physical exercise but rather a social promoter of the digital solution and the idea of a healthy lifestyle.

Returning to long-term goals and the possibility to adjust one’s goals, we found that only the most dedicated youths continued to use the technology after the Christmas break. Interviews revealed that these 4 participants had established their own long-term goals, and the participants without their own long-term goals had taken off the activity trackers and stopped using them. The 3 youths who used the activity tracker and accompanying app to the end of the project also expressed that the app not only motivated them to move regularly but it also motivated them to steadily increase their physical activity over the course of the project. For these youths, the digital technology in itself and their own long-term goals were sufficient motivators. It would be interesting for future research to study if, how, and to what extent long-term goals for youths in need of social care can be supported by digital technology.

In this study, the studied youths all expressed that being aware of one’s sleeping patterns was helpful. Half of the studied group improved their sleeping habits, whereas the other half did not. It was identified that the technology in itself did not generate solutions or provide sufficient information that could change the poor sleep habits of some of the youths. The challenge to figure out what was needed on a practical level to establish a positive change was too great for the youths themselves. Going to bed earlier did not necessarily lead to more sleep if there were more complicated reasons behind the sleeping problems. The conclusion was that supplementary practical support by adequate professionals is needed. There exist mobile phone apps that have been developed by researchers aimed to improve sleeping habits [37], and these are also related to intervention programs [38]. The latter pioneer study presents results from a 4-week field study with 12 participants. Their results demonstrate that a very low effort, recommendation-based peripheral display can be an effective method for improving awareness of healthy sleep habits. The accompanying app included an option to specify judged need of sleep, which then was compared against measured sleeping results. If the problem with sleep actually was because of just having bad habits, then the activity trackers and the accompanying app were of help to visualize, monitor, and motivate change.

In previous research, a criticism has been raised regarding the lack of involvement of health professionals during the development of technology, the monitoring of its use, and the feedback given to users [22,23], and thus, it makes sense to clarify our situation in this respect. In the project, the professional social workers were involved as active users. The staff did not take any level of responsibility for the project’s proceeding nor did they systematically test the app in their treatment process. This was because the staff had had a bad experience with taking responsibility in a previous externally introduced project. In this study, the staff chose to use the activity tracker and the accompanying app for reasons of curiosity. The staff also reflected over the results and the technology’s potential usefulness related to their clients at the end of the study. The caregivers found that the activity tracker and its accompanying app opened up for new type of talks about healthy exercise and sleep habits and health with the youths. Hence, the study opens up a new topic for discussion concerning whether the technology might be a valuable tool to help professionals within social work to help youths to achieve better structure and lifestyle habits.

### Conclusions

Motivation apps and their implementation for preventing health-related problems within social work have been identified as a gap in research. To contribute to this area, this research project was based on the following question: *What effects will the studied youths experience in relation to exercise and sleep as a result of using a wearable activity tracker and its accompanying app?* The youths’ daily movement was high as they on average walked more than 10,000 steps a day, which corresponds to walking about 8 km daily. The reminder function in the app was useful for some youths because it made it easier to remember to go to sleep on time, which affected the next day in a positive manner. Furthermore, the youths who did not succeed in establishing good sleep and movement habits still expressed that they had gained a better awareness of themselves regarding sleep and exercise.

In general, all youths expressed that they were motivated by the technology and the social attention that followed from its use and from participation in the project. The instant graphical feedback and sharing of information played a crucial role here. When taking a closer look, we could see that the motivation came from different sources. Social attention, being a member of a social group, and the friendly competition motivated all the studied youths. When the friendly competition changed to real competition, however, some of the youths felt negative stress. Half of the studied youths had the additional motivating factor of having established long-term goals. The youths who had not established long-term goals stopped using the device during the holiday break when the social context was lost, whereas those who had established long-term goals continued to use the activity trackers and app after the break and to the end of the project. The increased awareness of one’s own sleeping patterns did not by default generate motivation for all youths because it was hard for the youths to know what was needed to change their bad sleeping habits. Here, supplementary practical support by trained professionals is needed. When it comes to the staff, they emphasized that the activity tracker and accompanying app opened up for new types of talks about habits and health issues with the youths. They expressed that the device and app was a useful instrument for making visible the youths’ habits, structures, and patterns that might influence health, and this enabled the establishment of good discussions. The staff saw great potential for this technology to assist in their work to create better structure and patterns in everyday life for the youths under their care.

In summary, increased personal awareness, support from social workers, and friendly competition all supported the establishment of health goals for the youths. Although both instant graphical feedback and sharing information through friendly competition had a positive impact, these were not influential beyond the moment and social context. Although this was a short study, having long-term goals was found to be the most powerful factor for influencing the youths to keep on using the app during the study. It is still difficult to predict what the long-term value of the awareness of one’s own activity and sleeping habits will be, and longitudinal studies are needed here. More research is also needed relating to how activity trackers can support personal long-term health goals for youths and what influence and role professional caregivers can have here.

## Abbreviations

HBV: Home for Care or Living (In Swedish, HVB_hem: Hem för vård eller boende)
mHealth: mobile health
